# Computational blood flow simulations in Kawasaki disease patients: Insight into coronary artery aneurysm hemodynamics

**DOI:** 10.21542/gcsp.2017.29

**Published:** 2017-10-31

**Authors:** Noelia Grande Gutierrez, Andrew Kahn, Jane C. Burns, Alison L. Marsden

**Affiliations:** Cardiovascular Biomechanics Computation Lab, Stanford University, Stanford CA 94305-5428, USA

## Introduction

Coronary artery aneurysms (CAA) as a result of Kawasaki disease (KD) create abnormal flow conditions that can ultimately lead to thrombosis, with associated risks of myocardial infarction and sudden death^1–3^. The primary long-term clinical decision required for KD patients with aneurysms is whether to treat with anticoagulation therapy. Current clinical guidelines recommend CAA diameter ≥ 8 mm or Z-score >10 as the criterion for initiating systemic anticoagulation therapy^4,5^. In general, these aneurysms cause regions of flow stagnation, but the complexity of their geometry including changes in diameter, tortuosity and even proximal and distal stenoses make it difficult to evaluate thrombotic risk and predict patient outcomes based solely on a single anatomical measurement taken from image data, usually the maximum aneurysm diameter.

Combining modern tools for medical image processing and computational fluid dynamics now allows us to augment the information routinely extracted from imaging studies such us Computed Tomography Angiography (CTA) or Cardiac Magnetic Resonance Imaging (CMRI). We can now construct 3D anatomical models of the coronary arteries of individual patients and use numerical methods to solve the fluid mechanics equations that govern blood flow. Pressure and velocity fields computed in these 3D models can then be used to derive other hemodynamic quantities of interest that may be of clinical relevance.

This paper summarizes the current state of the art of imaged-based computational fluid dynamics in Kawasaki disease patients and presents some examples that illustrate the potential added value of these tools in the clinical setting.

## Background

Data from previous studies suggest insights from hemodynamics may be useful in developing more specific risk assessment tools for KD patients with aneurysms^6,7^. Studies have reported low blood flow velocities and areas of flow stasis in aneurysms via invasive methods, such us Doppler flow wire measurements^8,9^ and have related these findings with the risk of thrombus formation^8^. In addition, recent patient-specific modeling and computer modeling of blood flow in KD patients showed that hemodynamic parameters may offer a promising approach for identifying aneurysmal regions at higher risk of thrombosis^6,7^. Results suggest that thrombotic risk assessment by means of hemodynamic parameters such as wall shear stress (WSS), oscillatory shear index (OSI) or particle residence time (PRT) may be superior to using diameter alone. A recent study on CAA assessment using Transluminal Attenuation Gradient (TAG) analysis in KD patients also supports the hypothesis that hemodynamic quantification may be superior to using CAA diameter measurements suggesting TAG may encode hemodynamic information not available from anatomy alone^10^.

## Methods

Patient-specific modeling and simulation is performed using the SimVascular^11^ open source software, with patient specific models constructed from CTA or CMRI data. In particular, the aorta and the main coronary branches are modeled to include as much detail as the image data quality allows. New image acquisition sequences produce high-resolution images enabling realistic reconstruction of the coronary tree. In this paper we have included three KD patients who underwent clinically indicated computed tomography angiogram (CTA) and were retrospectively enrolled in the simulation study. Two of these patients had CAAs and one had normal coronary arteries and served as control. This study was approved by the Institutional Review Board at the University of California San Diego and written subject consent or assent and parental consent were obtained as appropriate for the imaging and simulation studies. CTAs used for the patient-specific modeling were obtained on a CT750 HD 64-slice CT scanner (GE Healthcare, Milwaukee, WI) at the University of California San Diego.

To solve the fluid equations we discretize the domain into a finite element mesh. In this case, linear tetrahedral finite elements are used with a boundary layer mesh to accurately solve the fluid field near the wall. Also, local mesh refinement in the aneurysmal regions is used to ensure convergence in hemodynamics quantities in these regions of interest. Resulting meshes for KD models typically range from 1.5 to 4.5 million elements depending on the size of the patient and the number and size of CAAs.

The SimVascular 3-D finite element solver is used to solve the time-dependent Navier–Stokes equations for blood flow. Blood is modeled as an incompressible Newtonian fluid with a density of 1.06 g/cc and dynamic viscosity of 0.04 dyn/cm^2^. To prevent divergence due to backflow, additional stabilization terms are used at the outlet nodes, acting only during periods of flow reversal^12^.

**Figure 1.**
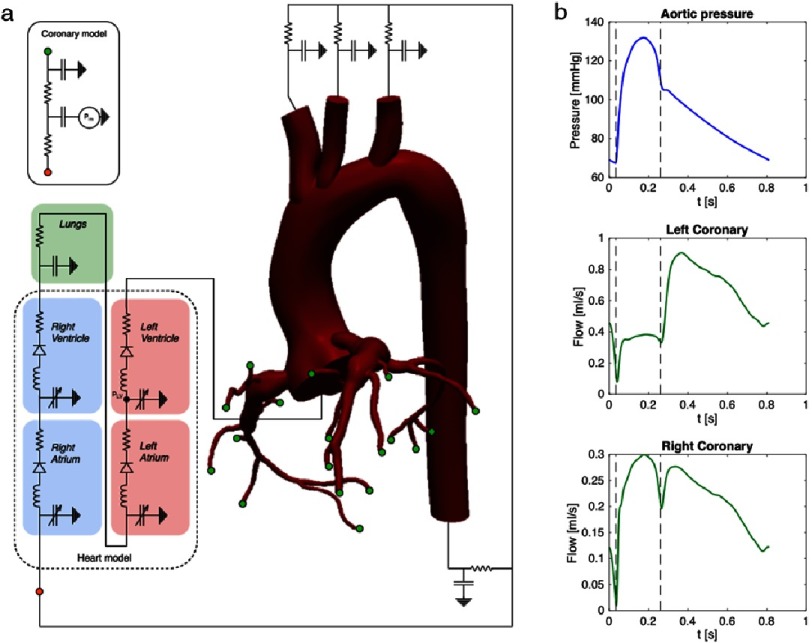
(a) Schematic of the closed loop lumped parameter network, which makes an analogy to electrical circuits. The Navier–Stokes equations are solved using a finite element method in the 3D domain and this is coupled numerically to a heart and downstream vascular resistance model, which accurately captures the coronary physiology. (b) Aortic pressure and coronary flow waveforms produced by the closed loop LPN simulation framework.

Coronary flow is notoriously challenging to model because it is out of phase with aortic flow due to myocardial contraction. During systole, the distal coronary resistance increases substantially due to increasing intra-myocardial pressure resulting from myocardial contraction. The complex interaction between coronary flow, aortic flow, and intra-myocardial pressure requires specialized boundary conditions to capture this complex physiology in the numerical model. A closed-loop, Lumped Parameter Network (LPN) is used to model the heart and vascular boundary conditions, and coupled numerically to the 3D flow solver using a semi-implicit scheme^13^. Closed-loop, LPNs have been used extensively by our group and others, particularly for single ventricle and coronary artery bypass graft (CABG) patients^14–21^ and this framework can also be applied to CAA simulations in KD patients. A schematic of the electrical analogy used to represent the coronary boundary conditions and the closed loop configuration in a KD patient is shown in Figure 1.

The heart model and outlet boundary condition parameters of the LPN are calculated to match the clinical and physiologic data of each patient using an automated parameter estimation method^22^. Parameters are normalized to patient stroke volume and body surface area to facilitate the automatic tuning process and match patient clinical targets consistently.

The current method included in SimVascular for deformable wall simulations, or fluid structure interaction (FSI), is the coupled momentum method, which simulates radial wall deformation using a membrane approximation and small deformation theory^23^. We assign wall material properties (i.e., Young’s modulus) and wall thickness, with variation through the model based on previous work^14,24^, allowing for appropriate material property values in different vessels such as the aorta and coronary arteries. If patient-specific information is available for arterial stiffness or thickness, as measured from intravascular ultrasound (IVUS) or optical coherence tomography (OCT), it can also be included in the model, though these data are typically not available for KD patients.

Inclusion of a closed loop LPN that produces physiological pressures together with appropriate material properties increases the accuracy of the FSI predictions. Our multi-scale framework allows not only the computation of the 3D fluid velocity and pressure fields but also provides physiologically realistic waveforms for the aortic pressure and coronary flow (Figure 1). Taken together, these methods enable physiologically realistic models of KD hemodynamics, which is a prerequisite for eventual use as a clinical tool for thrombosis risk prediction. Previous studies have validated simulation predictions of aneurysm hemodynamics against phase contrast MRI data using 3D printed *in vitro* models^25^.

To provide direct comparison of the CAA diameter effect on the local hemodynamics we virtually morphed a KD patient coronary artery model to simulate aneurysms with a range of aspect ratios, keeping the maximum aneurysm diameter fixed. The morphing of the aneurysm was done based on the patient-specific coronary artery centerline and diameter distribution was calculated using spline interpolation given the CAA length and maximum diameter. Geometric parameters of CAAs generated for this study are included in Table 1.

**Table 1:** Geometric and hemodynamic parameters for virtually generated coronary artery aneurysms. Time averaged wall shear stress (TAWSS) is the average wall shear stress value in the aneurysm averaged through one cardiac cycle. Area_WSS4_ and Area_WSS1_ represent the percentage of the aneurysm surface area exposed to TAWSS < 4 dynes/cm^2^ and TAWSS < 1 dynes/cm^2^ respectively.

Case ID	D_max_ [mm]	Length [mm]	Aspect ratio	TAWSS [dynes/cm^2^]	Area_wss4_ [%]	Area_wss1_ [%]
a.1	8	20	2.50	5.15	72.73	17.49
a.2	8	40	5.00	3.54	83.43	15.80
b.1	7	40	5.71	4.13	75.02	16.88
b.2	7	80	11.43	2.25	88.16	21.30

**Figure 2.**
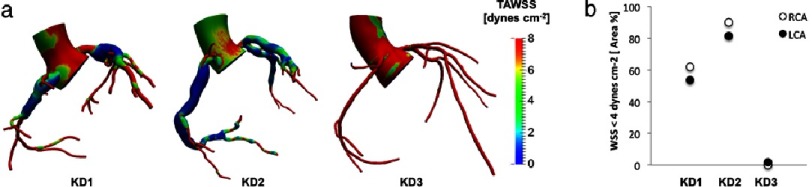
(a) Time average wall shear stress (TAWSS) distribution and (b) percentage area exposed to low wall shear stress in KD patients with CAAs (KD1, KD2) and with normal coronary arteries (KD3).

## Results of computational blood flow simulations in KD patients

Computational blood flow simulations have been widely used during the last decade for surgical planning and evaluation of disease progression in cardiovascular disease. In particular, they have been extensively applied in congenital heart disease^19,20,26,27^, abdominal and cerebral aneurysms^28–30^ and coronary artery bypass graft (CABG)^14,21^. The study by Sengupta et al.^6^ presented the first patient-specific hemodynamic simulations in a KD patient, reporting that the presence of an aneurysm in the proximal coronary artery led to flow recirculation, reduced wall shear stress within the aneurysm, and high wall shear stress gradients at the neck of the aneurysm. A follow-up study by Sengupta et al. using computational simulations in a cohort of five KD patients with CAA and a normal control suggested that fusiform aneurysms may entail higher risk of thrombosis compared to saccular aneurysms^7^ and that hemodynamic measures may more effectively stratify patient risk than diameter alone.

Figure 2 shows simulation results in three different KD patients. This example illustrates the effect of coronary artery geometry on local hemodynamics both qualitatively and quantitatively. Note that the largest diameter CAA does not produce the lowest WSS values in this case, a finding that is contrary to current opinion, which equates larger diameter with a higher probability of thrombosis. This supports the hypothesis of recent simulation studies reporting that risk of thrombosis may not directly correlate with maximum aneurysm diameter.

**Figure 3.**
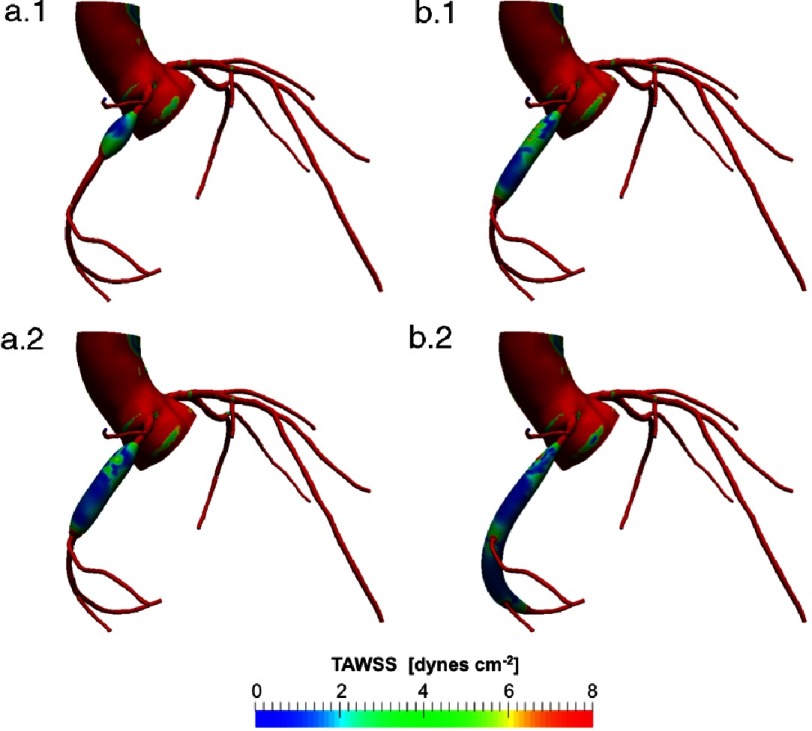
Comparison of time average wall shear stress (TAWSS) distribution on virtually morphed CAAs (Length/D_max_ [mm]): a.1) 2.5 (20/8), a.2) 5 (40/8), b.1) 5.71 (40/7), b.2) 11.43 (80/7).

For the virtually morphed CAAs all other simulation parameters were held constant. Therefore, all changes seen in the coronary artery hemodynamics are induced by changing the geometry in the coronary flow. According to a diameter or Z-score classification, all these aneurysm would represent the same risk of thrombosis. However, examining wall shear stress or flow stagnation enables us to rank the aneurysms according to thrombotic risk in a different way. In particular, looking at the example in Figure 3 we can infer that WSS correlates with aspect ratio (Table 1). Cases a) and b) present aneurysms with a maximum diameter of 8 and 7 mm, respectively, but different aspect ratios (Table 1). This example highlights the importance of a hemodynamic assessment in CAA; abnormal WSS values are found in case b) even though the maximum diameter is below the 8 mm cutoff. We note that in this example we modified only one shape parameter, the aneurysm length, leading to intuitive changes in hemodynamics. Aneurysms found in KD patients are rarely as simple; they typically have complex geometry with multiple lobes and asymmetry with respect to the coronary artery centerline. In these cases for which geometry classification is not straightforward, computational simulations can be particularly useful in producing quantitative hemodynamic metrics.

## Discussion

Patient-specific modeling provides a non-invasive means of obtaining the unique hemodynamic characteristics of each CAA and therefore quantifying abnormal coronary flow for individual patients. The use of computational modeling in KD patients may ultimately lead to individually tailored therapy based on quantitative, patient-specific hemodynamic parameters.

In addition, data obtained from computational simulations provide a local description of the hemodynamics, which could not only allow us to better determine which patients would benefit from anticoagulation treatment to prevent coronary artery thrombosis, but also to better plan potential coronary interventions. Results from simulations could be a valuable input for surgical planning such as coronary artery bypass graft surgery, providing the optimal graft location to optimize hemodynamic conditions in the native coronary arteries, CAAs, and grafts.

While patient-specific modeling can provide a wealth of hemodynamic information, there are still numerous challenges associated with performing these simulations in routine clinical practice, as they require labor-intensive model construction and high-performance parallel computing. However, the steady increase of computer power and more efficient numerical methods may improve the turn-around time for these computational models. Also, incorporating machine learning and reduced order modeling will likely contribute to significant increases in efficiency, enabling computational simulations to be performed for routine clinical use.

More analysis is needed in future studies to determine statistical correlations between hemodynamic quantities of interest and patient outcomes such as development of thrombosis. We must evaluate the relative contribution of each hemodynamic parameter to the development of coronary thrombosis on a per-patient and per-vessel basis. In particular, wall shear stress levels, integrated high and low shear stress exposure, wall shear stress gradients and particle residence times, should be correlated with outcome data. Challenges of multi-center data collection will need to be overcome to assemble a large enough database of outcomes in this relatively rare disease. These findings could ultimately lead to the development of a novel risk stratification index that could identify individuals at risk for adverse cardiovascular events with sufficient predictive accuracy to become a clinical tool.
